# Halophilic Archaea: Life with Desiccation, Radiation and Oligotrophy over Geological Times

**DOI:** 10.3390/life5031487

**Published:** 2015-07-28

**Authors:** Helga Stan-Lotter, Sergiu Fendrihan

**Affiliations:** 1 Division of Molecular Biology, University of Salzburg, Hellbrunnerstr. 34, Salzburg 5020, Austria; 2 Romanian Bioresource Centre and Advanced Research, Aleea Istru 2C, blocul A 14 B, scara 8, apartment 113, Bucharest 061912, Romania; 3 Institute of Microbiology, Virology and Parasitology, Vasile Goldis Western University Arad, 94-96 Revolutiei Blvd., Arad 310025, Romania; E-Mail: ecologos23@yahoo.com

**Keywords:** *Halococcus* species, *Halococcus salifodinae*, Haloarchaea, long-term survival, halomucin, polyploidy, ancient salt deposit, extraterrestrial halite

## Abstract

Halophilic archaebacteria (Haloarchaea) can survive extreme desiccation, starvation and radiation, sometimes apparently for millions of years. Several of the strategies that are involved appear specific for Haloarchaea (for example, the formation of halomucin, survival in fluid inclusions of halite), and some are known from other prokaryotes (dwarfing of cells, reduction of ATP). Several newly-discovered haloarchaeal strategies that were inferred to possibly promote long-term survival—halomucin, polyploidy, usage of DNA as a phosphate storage polymer, production of spherical dormant stages—remain to be characterized in detail. More information on potential strategies is desirable, since evidence for the presence of halite on Mars and on several moons in the solar system increased interest in halophiles with respect to the search for extraterrestrial life. This review deals in particular with novel findings and hypotheses on haloarchaeal long-term survival.

## 1. Introduction

Halophilic Archaea (or Haloarchaea) thrive in environments with salt concentrations approaching saturation, such as natural brines, alkaline salt lakes, the Dead Sea and marine solar salterns. They have also been found worldwide in rock salt deposits of great geological ages, from the Pliocene (5.3–1.8 million years) up to the Silurian (419 million years) (see the overview in [1]).

The first cultivations of halophilic microorganisms from Permian salt sediments (about 250 million years old) were reported in the 1960s [2,3] and raised considerable skepticism. This critical view has not changed, since contamination issues are being suspected and an age of millions of years for a viable microorganism is difficult to imagine. From the 1990s onward, sequences of small ribosomal RNA (16S rRNA) genes and, increasingly, full taxonomic descriptions of cultivated isolates revealed novel species of Haloarchaea in ancient rock salt [1,4,5,6,7,8,9,10,11,12,13,14,15]. There is now a growing body of evidence that halophilic prokaryotes, many of them Haloarchaea, are able to survive in rock salt, most likely within brine-filled fluid inclusions [13,16,17,18], for very long periods of time. These findings raise questions about the strategies and mechanisms that may allow such longevity. The halophilic microorganisms in ancient evaporites have experienced hypersaline environments, which were characterized by extremely high ionic strength, elevated temperatures and probably high levels of UV radiation. Periodic evaporation of water had taken place, which caused the concentration of salts and exposure of cells to desiccation. Furthermore, nutrients were depleted, and over time, the environment became oligotrophic, which denotes conditions of low availability of carbon, phosphate and nitrogen. A well-known survival modus in response to such stressors are spores, which are dormant forms of several bacteria and eukaryotes with high resilience to environmental extremes. However, genome sequences suggested that Haloarchaea (and Archaea in general) do not produce spores [19]. The occurrence of other types of haloarchaeal resting states, such as cysts, has been discussed [20], but has not yet been demonstrated unequivocally.

This review presents newer data on some strategies of Haloarchaea for coping with multiple stresses of high salt (or low water activity), oligotrophic conditions, long-term desiccation and resistance to irradiation. The occurrence of polyploidy in many prokaryotes is a recently-discovered property that provides a previously unexpected basis for several modes of resistance [15,21,22,23,24,25], as is discussed below. Finally, a consideration of these findings with respect to the possibility of halophilic life on other planets or moons is provided.

## 2. Specific Strategies of Haloarchaea

### 2.1. Low Water Activity and Desiccation

Microorganisms that are exposed to a low water activity environment have to apply strategies to avoid water loss by osmosis. The term water activity (a_w_) is widely used to define the availability of water in a particular environment. Water activity denotes the amount of water available for the hydration of materials; a value of 1.0 indicates pure water. The addition of solutes lowers a_w_ to values below 1.0. The a_w_ of saturated salt lakes, for example, is 0.75, which is the typical environment of numerous halophiles. Several halophilic microorganisms use counter-balancing levels of inorganic ions (usually KCl) to achieve osmotic stability; others produce or accumulate compounds of low molecular mass, the so-called compatible solutes (for reviews, see [26,27]).

*Haloquadratum walsbyi* is truly unique among Haloarchaea and even prokaryotes, since its cells are square and flat, forming often extended sheets [28]; its GC content is much lower (48%) than that of other Haloarchaea (60%–70%); its gene density is quite low (76%); and it contains halomucin, a very large protein [29,30]. Halomucin of *Hqr. walsbyi* strain HBSQ001 consists of 9159 amino acids, and its sequence and domain organization are similar to those of animal mucins, which are known to protect various tissues against desiccation [29]. Halomucin is secreted and apparently surrounds the cells as a water-enriched cloud of protein, as was deduced from specific antibody staining [30,31]. Due to the fact that it is highly glycosylated and sulfated, it is thought to form a water-rich capsule around the cells and to protect against conditions of desiccation or extremely low water activity [29]. An additional role of halomucin might be a barrier against phages [31]. *Hqr. walsbyi* has so far not been found in ancient halite samples [1]. In view of its nearly global distribution—Israel, Peru, Spain, Tunisia, Turkey, Australia—it was suggested that *Hqr. walsbyi* might survive in fluid inclusions of halite crystals during its dispersal by wind or migratory birds [32]. In any case, *Hqr. walsbyi* is a dominant microorganism in highly salty ecological niches [30], which are known to support biological growth on Earth, and these defense strategies appear so far unique.

### 2.2. Oligotrophic Environments and Starvation

Oligotrophs are organisms that can live in nutrient-poor environments. They grow slowly and have low rates of metabolism, which lead to low population densities. Oligotrophs occur in deep oceanic sediments, caves, glacial and polar ice, deep subsurface environments, ocean waters and leached soils. The concentration of total organic carbon in those environments is in the range of one to a few milligrams per liter [33]. Such environments were long considered to be “deserts” for life, and microbes, which were sometimes seen in the microscope, were assumed to be dead, dormant or at least severely starved bacterial cells [33]. Only during the last few years was it recognized that most of these microbes are perfectly alive, metabolizing and ready to grow when given the chance [33]. Adaptation to nutrient limitation by many oligotrophic microorganisms consists of increasing the surface-to-volume ratio, which increases the capacity for nutrient uptake relative to cell volume. This is often apparent as the formation of miniaturized cells. In this way the organism’s capacity to scavenge available energy-yielding substrates will be increased [34]. A high surface-to-volume ratio is thus typical for many oligotrophic bacteria. The haloarchaeon *Haloquadratum walsbyi* manages the increase of surface area in a somewhat different way, namely by extremely flattening itself [35]. Its thickness is 0.1–0.5 μm, and it achieves thus what is probably the highest surface-to-volume ratio in the microbial world [29,35]. Whereas spherically-shaped microorganisms have to remain small, squares can become unlimitedly large, since the surface-to-volume ratio solely depends on their thickness [29]. The ability of *Hqr. walsbyi* for efficient phototrophic growth by spreading out the flat cells on the water surface, much like a molecular solar panel, is also unique among prokaryotes [29] and represents an adaptation to the oligotrophic environment of hypersaline salterns. A novel finding of the miniaturization of some Haloarchaea in fluid inclusions as a response to low water activity [36] is described in more detail below.

Marine bacteria are known to grow on external dissolved DNA [37], using it as a source of carbon, nitrogen and phosphorus. Recently, it was shown that the archaeon *Haloferax volcanii* is also able to use external DNA as a nutrient source and, as it turned out, even internal DNA as a source of phosphate [25]. The latter ability is possible due to its polyploid state. These findings have some implications on the survival of Haloarchaea in fluid inclusions. Microscopic algae, such as *Dunaliella* sp., were detected in fluid inclusions, together with Haloarchaea [17]. Their lysis products, perhaps together with those of Haloarchaea, containing DNA and other molecules, might be sufficient to sustain a minimal maintenance metabolism, including DNA repair, of intact cells.

### 2.3. Sphere Formation in Fluid Inclusions

**Figure 1:**
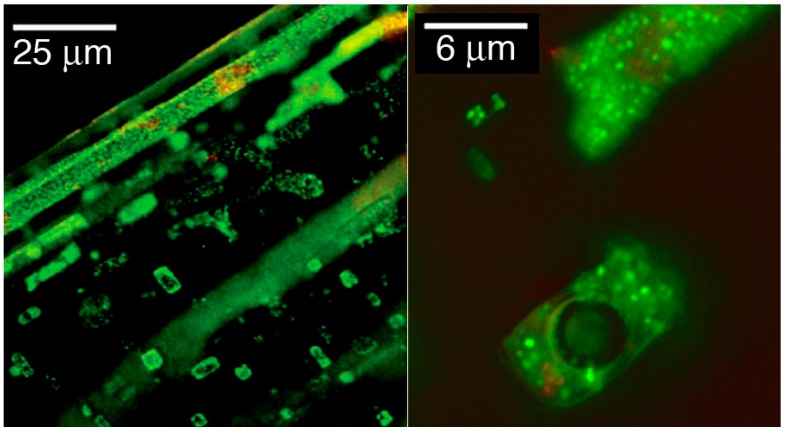
Localization of pre-stained cells of *Halobacterium salinarum* NRC-1 in fluid inclusions of laboratory-produced halite. Low magnification (left) and higher magnification of several fluid inclusions (right). Cells were stained with the LIVE/DEAD^®^ *Bac*Light^™^ bacterial viability kit prior to embedding in halite.

Fluid inclusions are present in natural halite [38] and were considered early as possible habitats for halophilic microorganisms [39]. From a fluid inclusion in a 97,000-year-old halite crystal from Death Valley, a strain of *Halobacterium salinarum* was isolated [9]. Cells within fluid inclusions can be visualized in laboratory-grown halite, as was demonstrated for the non-halophilic *Pseudomonas aeruginosa* [40] and several Haloarchaea [41,42,43]. Cells of a rod-shaped morphology, such as strains of *Halobacterium salinarum*, were converted to small roundish forms within the fluid inclusions, as shown in Figure 1. Recently, small particles of about 0.4 µm in diameter were imaged by microscopy directly within fluid inclusions of 22,000–34,000-year-old salt bore cores and, following successful culturing, identified as Haloarchaea [13,16].

**Figure 2:**
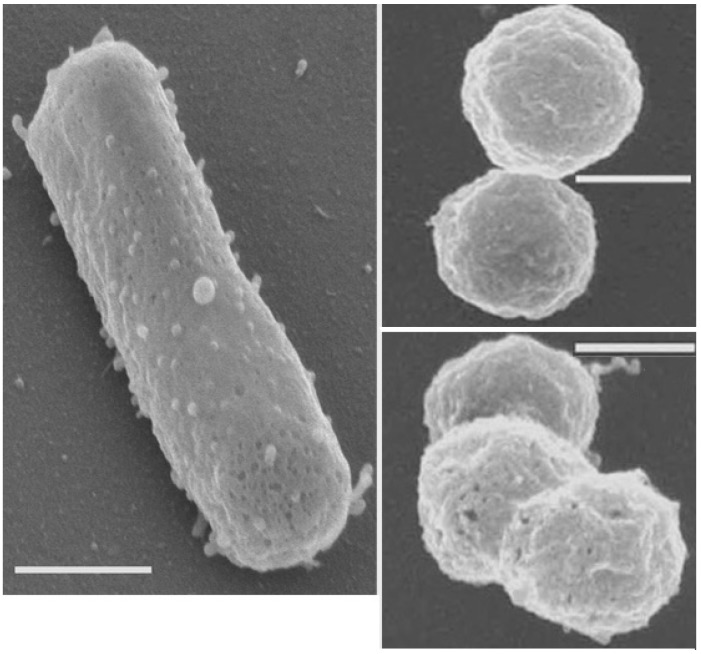
Scanning electron micrographs of a rod (left panel) and spheres (right panels) of *Halobacterium salinarum* NRC-1. Spheres had formed within fluid inclusions of laboratory-grown halite and were obtained after dissolution of salt crystals. Bars, 270 nm. Photographs taken by Chris Frethem, University of Minnesota.

The small particles are thought to represent a form of miniaturization. We examined the spherical particles in laboratory-produced fluid inclusions (Figure 1) in detail and showed that sphere formation is apparently a response to low external water activity (a_w_) of several haloarchaeal species [36]. Rod-shaped cells of *Halobacterium* species gradually converted to small spheres (see Figure 2) upon a decrease of the external a_w_ to less than 0.75. From one rod, three to four spheres were formed. The diameter of the spheres was 0.40 ± 0.02 µm [36].

An explanation for the spherical particles, which were observed in fluid inclusions of ancient and well-dated halite [13,16], could thus be the transformation from former rod-shaped Haloarchaea to roundish forms as a response to lowered external a_w_. The metabolic activity of enclosed microbial cells might possibly alter a_w_ within a fluid inclusion, although this has never been tested. We obtained some indirect experimental evidence for this notion by the rapid production of spheres in the laboratory upon exposing haloarchaeal cells to buffered 4 M LiCl solution, which exhibits an a_w_ of about 0.73 [36]. Exposure of haloarchaeal rod-shaped cells turned those cells immediately into spheres. The haloarchaeal spheres stayed viable for years, when kept in buffered 4 M NaCl and, following the addition of nutrients, proliferated into normal rods. An initial biochemical characterization showed that spheres contained an about 50-fold lower content of ATP, compared to rods [36]. It seems likely that diminishing the surface-to-volume ratio is part of the strategy for long-term survival, as the roundish particles observed in fluid inclusions of ancient halite crystals suggested [13,16]. It was pointed out by Zerulla and Soppa [24] that the conversion of one cell to several spheres would be impossible for monoploid species, because only one of the 3–4 spheres would obtain a copy of the chromosome. The confirmed counts of spheres formed from cells can thus serve as an indirect proof for the occurrence of polyploidy in several Haloarchaea [36].

### 2.4. Resistance to Radiation

Remarkable resistance to ionizing radiation has been reported for *Halobacterium salinarum*, which showed a D_10_ value (the dose of radiation in Gray (Gy) that reduces the survival of a population by 90%) of 5 kGy, which is of a similar order of magnitude as that of the archaeon *Thermococcus gammatolerans* (6 kGy) and of the bacterium *Deinococcus radiodurans* (12 kGy) [44,45]. Two irradiation-derived mutants of *Hbt. salinarum* displayed even D_10_ values close to that of *D. radiodurans* [46]. Both desiccation and irradiation result in double-strand breaks of DNA. The connection between resistance to these treatments had been established previously [47], and the survival of such conditions may have a universal basis. Recovery from irradiation, as well as desiccation occurred quickly in *Hbt. salinarum* [44]. An elegant explanation for the mechanism underlying this behavior is the presence of several chromosomes and the ability to regenerate intact chromosomes from scattered fragments [22]. Overlapping genomic fragments are a prerequisite for this mechanism, and thus, it can only operate in oligoploid, such as *D. radiodurans*, or polyploid species, such as Haloarchaea [23].

Since not all polyploids are radiation and desiccation resistant, other factors are being sought for these resiliences. For example, Capes *et al.* [48] were screening nine complete haloarchaeal genomes and showed that nearly 800 protein clusters were present in all Haloarchaea, with a subset of 55, which were considered critical or essential for success in extreme environments. One protein, Ral (tucHOG0456) from *Halobacterium salinarum* NRC-1, was suggested to function in double-stranded DNA break repair and tolerance to desiccation and radiation. Webb and DiRuggiero [45] reviewed further studies on the potentially wide diversity of protective molecules and systems of radiation resistance in Haloarchaea and other prokaryotes.

Natural halite contains considerable background radiation from the isotope ^40^K, which is thought to limit the viability of spores in fluid inclusions to maybe 109 million years or less [49]. These considerations were recently taken into account for projecting the chances of microbial life on Mars [50]. However, we suggest to follow the argumentation by Soppa [23] considering the often assumed chemical instability of DNA of less than about 100,000 years, which may be valid for monoploid species, but is not applicable to polyploid species. As long as maintenance metabolism is possible, DNA damage in some copies of the chromosome can be repaired using the remaining intact copies of polyploid species as templates [22,23].

Interestingly, three haloarchaeal species have been isolated recently from 38–41 million-year-old salt deposits, and all three were shown to be polyploids, in agreement with the prediction that polyploidy enables long-term survival [15].

## 3. Extraterrestrial Halite

If halophilic prokaryotes on Earth can remain viable for very long periods of time, then it is reasonable to consider the possibility that viable microorganisms may exist in similar subterranean salt deposits on other planets or moons. Extraterrestrial halite has been identified in Martian meteorites [51], in the Murchison and other carbonaceous meteorites [52] and in the Monahans meteorite, together with sylvite (KCl) and water inclusions [53]. Postberg *et al.* [54] found that about 6% of the ice grains from the plumes of Saturn’s moon Enceladus were quite salty, containing roughly 1.5% of a mixture of sodium chloride, sodium carbonate and sodium bicarbonate. Recent images from the Mars Reconnaissance Orbiter showed evidence for seasonal emergence of liquid flows down steep rocky cliffs in summer, which would be consistent with briny liquid water emerging from underground reservoirs on Mars [55]. There is evidence for various brines on Jupiter’s moon Europa that are composed primarily of water and salts [56]. All of these discoveries make the consideration of potential habitats for halophilic life in space intriguing.

The apparent longevity of haloarchaeal strains in dry salty environments is relevant for astrobiological studies in general and, in particular, for the search for life on Mars. A detailed investigation of the mode of sphere formation and their properties could lead to insight for how Haloarchaea inside natural fluid inclusions, where low a_w_ is prevalent, may survive. Fluid inclusions with high salt concentrations from a dated meteorite show that water trapped in halite can be preserved for billions of years [53]. Thus, such studies may also be useful for the design of experiments aimed at the detection of potential extraterrestrial forms of microbial life in sediments of great geological age.

## 4. Conclusions

Haloarchaea can survive extreme desiccation and starvation, sometimes apparently for geological time periods, *i.e.,* millions of years. The mechanisms that appear to be involved include dwarfing of cells, reduction of ATP, the formation of protective capsules and probably the production of dormant stages, such as small spherical particles. Polyploidy is an essential factor in several of these strategies.

Recent data suggested the presence of halite on Mars, as well as on several moons in the solar system. To increase the chances for finding extraterrestrial life, halite-containing regions should be considered, and the search should include a focus on very small particles, which might be living fossils.
